# Function-guided differences of arcuate fascicle and inferior fronto-occipital fascicle tractography as diagnostic indicators for surgical risk stratification

**DOI:** 10.1007/s00429-024-02787-3

**Published:** 2024-04-10

**Authors:** Leonie Kram, Axel Schroeder, Bernhard Meyer, Sandro M. Krieg, Sebastian Ille

**Affiliations:** 1 Department of Neurosurgery, Technical University of Munich, School of Medicine, Klinikum rechts der Isar, Munich, Germany; 2 Department of Neurosurgery, Heidelberg University Hospital, Ruprecht-Karls-University, Heidelberg, Germany

**Keywords:** Language mapping, Brain tumor, Diffusion tensor imaging-based tractography, Navigated transcranial magnetic stimulation, Aphasia risk stratification

## Abstract

**Background:**

Several patients with language-eloquent gliomas face language deterioration postoperatively. Persistent aphasia is frequently associated with damage to subcortical language pathways. Underlying mechanisms still need to be better understood, complicating preoperative risk assessment. This study compared qualitative and quantitative functionally relevant subcortical differences pre- and directly postoperatively in glioma patients with and without aphasia.

**Methods:**

Language-relevant cortical sites were defined using navigated transcranial magnetic stimulation (nTMS) language mapping in 74 patients between 07/2016 and 07/2019. Post-hoc nTMS-based diffusion tensor imaging tractography was used to compare a tract’s pre- and postoperative visualization, volume and fractional anisotropy (FA), and the preoperative distance between tract and lesion and postoperative overlap with the resection cavity between the following groups: no aphasia (NoA), tumor- or previous resection induced aphasia persistent pre- and postoperatively (TIA_P), and surgery-induced transient or permanent aphasia (SIA_T or SIA_P).

**Results:**

Patients with NoA, TIA_P, SIA_T, and SIA_P showed distinct fasciculus arcuatus (AF) and inferior-fronto-occipital fasciculus (IFOF) properties. The AF was more frequently reconstructable, and the FA of IFOF was higher in NoA than TIA_P cases (all p ≤ 0.03). Simultaneously, SIA_T cases showed higher IFOF fractional anisotropy than TIA_P cases (p < 0.001) and the most considerable AF volume loss overall. While not statistically significant, the four SIA_P cases showed complete loss of ventral language streams postoperatively, the highest resection-cavity-AF-overlap, and the shortest AF to tumor distance.

**Conclusion:**

Functionally relevant qualitative and quantitative differences in AF and IFOF provide a pre- and postoperative pathophysiological and clinically relevant diagnostic indicator that supports surgical risk stratification.

## Introduction

Preservation of functionality during resections of brain tumors plays a pivotal role in enhancing the patients’ quality of life and survival (Rahman et al. 2017). While maximal removal of language-eloquent tumors improves the overall prognosis (Brown et al. 2016), it simultaneously poses the risk of permanent loss of language abilities. This may comprise many expressive and receptive phonetic-phonologic, lexico-semantic, morpho-syntactic, or pragmatic language skills. Studies report an incidence of 17–100% of new postoperative deterioration of language function (Wilson et al. 2015). While a large proportion of these deficits are only transient in nature, up to 18% of patients show persistent and unrecovered aphasia even several months post-surgery (Sanai et al. 2008; Zetterling et al. 2020; Ilmberger et al. 2008; Caverzasi et al. 2016). Although any postoperative worsening can compromise the patient’s well-being and quality of life, exceptionally persisting and permanent deficits can tremendously impact overall emotional and social health, well-being, life quality, and overall survival rate (Krishna et al. 2021; Hervey-Jumper and Berger 2016). Consequently, it is crucial to provide reliable risk assessment preoperatively to allow an informed decision about the associated probability of postoperative worsening in language functionality. The latter depends on a dynamic and highly interconnected network frequently subdivided into a ventral and dorsal stream (Hickok and Poeppel 2004; Friederici 2017). Numerous studies based on lesion, imaging, stimulation and advanced computational machine learning approaches verified a crucial role of fasciculus arcuatus (AF) which constitutes an important part of the dorsal stream (Shams et al. 2023; Caverzasi et al. 2016; Tuncer et al. 2021). At the same time, the ventral stream can be subdivided into a direct and an indirect pathway (Duffau 2016). As the direct pathway, the inferior-frontal-occipital fasciculus (IFOF), connects frontal with occipital and superior parietal cortical areas (Sarubbo et al. 2013; Martino et al. 2010). Simultaneously, the indirect ventral pathway comprises the inferior longitudinal fascicle (ILF) and uncinate fascicle (UF) (Duffau et al. 2013). Still, studies associated predominantly damage of the direct pathway with the expression of language deficits, while indirect ventral pathway damages (ILF or UF) were compensable (Tuncer et al. 2021; Ius et al. 2011; Duffau et al. 2009; Mandonnet et al. 2007). Understanding and quantifying processes promoting or preventing aphasia may substantially support preoperative risk stratification and identifying practical and valuable individual targets for prehabilitation or rehabilitation approaches. Few studies thus far evaluated the utility of stimulation techniques for promoting the reallocation of language function before surgery to enable a more significant extent of resection without postoperative worsening or postoperatively for supporting the recovery of impaired language abilities (Rivera-Rivera et al. 2017; Poologaindran et al. 2022). Navigated transcranial magnetic stimulation (nTMS) is a non-invasive localization method that allows relating cortical anatomical areas to task-specific functions. Moreover, in combination with diffusion tensor imaging (DTI), it becomes feasible to investigate the functionally relevant subcortical network (Sollmann et al. 2016; Raffa et al. 2016). Thus, nTMS- and DTI-based language mappings are increasingly used for preoperative planning and neuro-navigated guidance of resections (Lefaucheur and Picht 2016; Ille et al. 2021a; Raffa et al. 2022).

Furthermore, the first studies assessed the suitability of these tractographies to stratify and quantify the risk of postoperative language worsening. For instance, Sollmann et al. (2020a) proposed that the distance between the tumor and common, functionally relevant language tracts may indicate the risk of postoperative transient or permanent language deficits. Additionally, Tuncer et al. (2021) showed that the preoperative infiltration of specific language tract components, as identified with DTI language tractography informed by anatomical seed regions, was related to permanent aphasia. At the same time, DTI allows quantitative parameters describing microstructural tissue properties (Winston 2012). The resulting fractional anisotropy (FA) value may indicate the white matter integrity by measuring the diffusivity consistency of water molecules within a specific region (Curran et al. 2016). Hence, DTI metrics are frequently used as microstructural markers to quantify lesion or degeneration-specific patterns in many different neurological disorders (Tae et al. 2018). As it stands, it remains widely unknown which factors can reproducibly and quantitatively predict postoperative deficits. The present study aimed to evaluate and quantify subcortical differences in functionally relevant language tracts between brain tumor patients with and without aphasia. To this end, we compared different quantitative and qualitative properties of function-based tractography of standard language network components across patients without and with transient or permanent postoperative language worsening. Since, moreover, the most significant proportion of language deficits are thought to be caused by the tumor (IJzerman-Korevaar et al. 2018), this study also differentiated between patients without and with preoperatively preexisting aphasia caused by the tumor or a previous resection.

## Material and methods

### Ethics

This study was approved by the institutional local ethics committee (reference number: 192/18S) and conducted in line with the guidelines of the Declaration of Helsinki. Informed consent was obtained from all individual participants included in this study.

### Patient selection

A post-hoc analysis of prospectively included patients who underwent preoperative nTMS-based language mapping between July 2016 and July 2019 at our university hospital was performed. Thus, this study cohort partly overlapped with a cohort included to ascertain the impact of nTMS-based language mapping on the postoperative outcome (Ille et al. 2021a). Of all consecutive cases, only patients fulfilling the following inclusion criteria were selected: 1) written informed consent, 2) at least 18 years of age, 3) no MRI or nTMS contraindications such as cardiac pacemakers or cochlear implants, 4) left-hemispheric, suspected language-eloquent glioma, 5) availability of a preoperative magnetic resonance imaging (MRI) sequence including diffusion tensor imaging (DTI) with 30 or 32 diffusion gradient directions, 6) clinical examination of the language status pre-, directly post- and three months postoperatively.

### Magnetic resonance imaging

All MRI sequences were acquired within the department of neuroradiology on a 3-Tesla scanner (Achieva dStream or Ingenia; Philips Healthcare, Best, Netherlands) with an 8- or 32-channel head coil. The standard protocol employed in our hospital for brain tumor imaging comprised at least a three-dimensional structural, T1-weighted gradient echo sequence (repetition time (TR)/ echo time (TE): 9/4 ms, one mm^3^ isovoxel covering the whole head) and a DTI sequence with 32 diffusion directions (TR/ TE: 5000/78 ms, b-values: 0 and 1000 s/mm^2^, spatial resolution: 2×2×2 mm^3^). These scans were typically acquired within a week before the nTMS mapping and repeated 48 h postoperatively.

### nTMS-based language mapping

An electric-field navigated nTMS system (NBS system 4.3 or 5.0, Nexstim Plc, Helsinki, Finland) was used to follow a routine and established language mapping protocol (Krieg et al. 2017, 2016). Patients were asked to name black and white drawings of everyday objects. At the same time, stimulation was applied over 46 predefined frontal, parietal, and temporal cortical sites at 5 Hz / 5 pulses, targeting each site three to six times. The intensity was set to 100 to 110% of the individual resting motor threshold as this defines the minimal necessary intensity to elicit a motor-evoked response in a hand muscle (Awiszus 2003). To tailor the image set to a patient’s abilities, before the nTMS application, two baseline trials were carried out. All items a patient could not name accurately and reproducibly were excluded. After the nTMS mapping session, examiners identified errors prompted by stimulation, such as no responses, performance errors, semantic paraphasia, or hesitations within post-hoc available video recordings of the respective stimulation session (Lioumis et al. 2012). Subsequently, all sites at which stimulation induced an error in task performance were marked as language-relevant and exported in three layers (peeling depth: 15, 20 and 25 mm) in DICOM format to guide tractography.

### Function-specific tractography

A deterministic tractography algorithm integrated into the surgical neuronavigation software Brainlab Elements (version 3.2.0.281, Brainlab AG, Germany) was employed for generating nTMS-based tractographies of the functional language network. Tractographies were derived for two time points to compare surgery-induced qualitative and quantitative changes for different functionally relevant language tracts pre- and directly post-surgery. For the preoperative tractography, preoperative individual structural T1-weighted images, the corresponding DTI sequences, and the nTMS-positive language-relevant cortical sites derived from the preoperative language mapping were aligned and fused with Brainlab Elements Rigid Image Fusion (Brainlab AG, Germany) based on a linear co-registration algorithm (Gerhardt et al. 2019). For the postoperative tractography, postsurgical T1-weighted and DTI sequences were used and again first aligned and fused on the basis of the system-integrated Rigid Image Fusion. As the patient’s general and medical condition directly post-surgery do typically not allow for a reliable nTMS language mapping, we fused the preoperative nTMS-positive language sites with the new postoperative imaging sequences. This approach has been implemented in previous studies (Negwer et al. 2018; Ille et al. 2018). To additionally control for the accuracy of this alignment, we additionally fused the pre- with the postoperative structural T1-weighted sequence. Since previous studies showed that even for pre- and intraoperative MRI data, for which a high extent of brain shift is frequently expected, the extent of this shift on a subcortical level was only around 1 mm (Ille et al. 2021b), a rigid fusion of both structural MRI sequences was performed. By overlaying both anatomical image sequences, the localization of the nTMS-positive language sites could be checked in reference to surrounding anatomical structures. An example of this additional qualitative control step is provided in Appendix A.

**Fig. 1:**
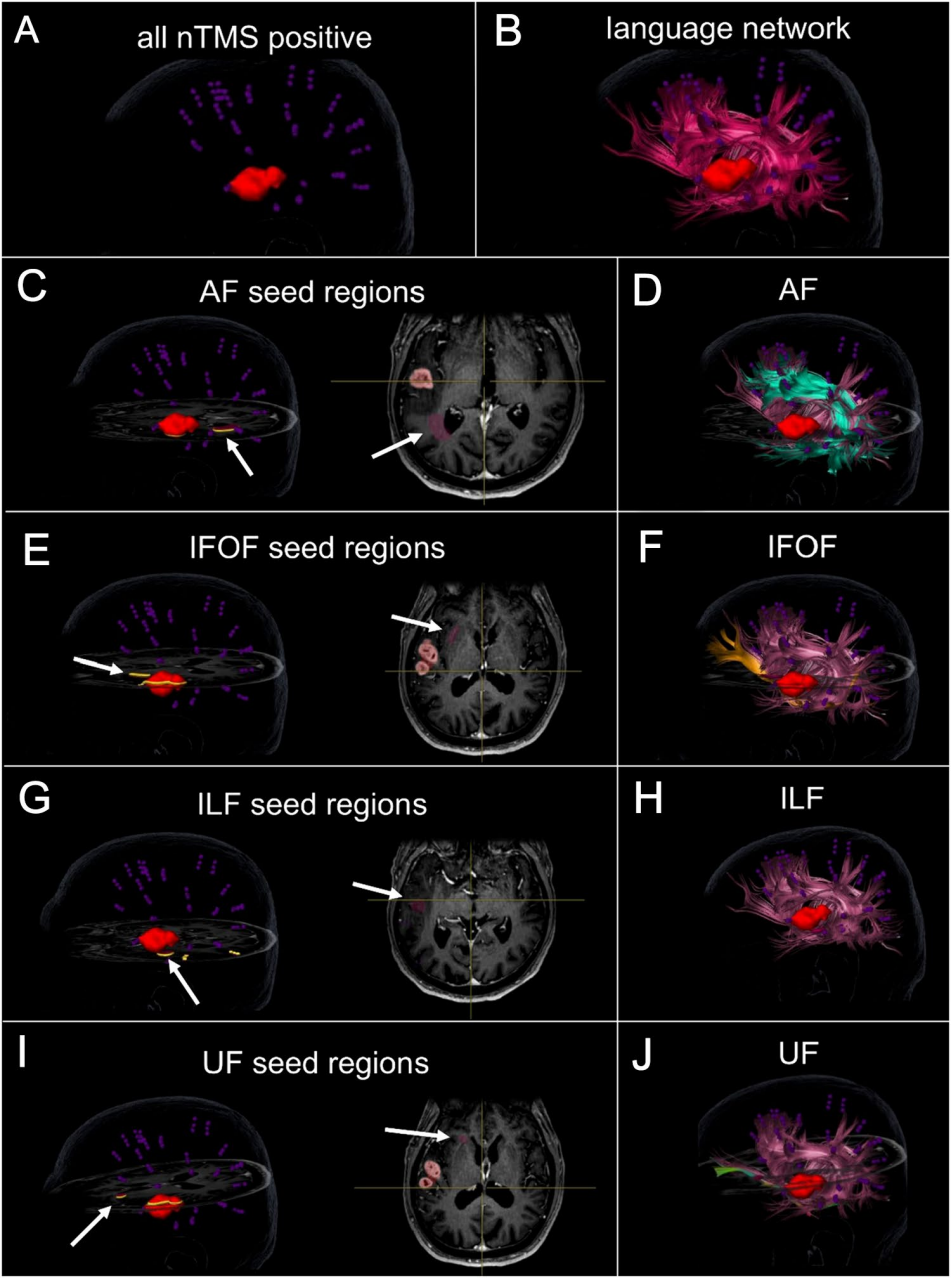
Overview of the tractography process for an exemplary patient case. All nTMS positive points (**A**) which were used to reconstruct the functional left-hemispheric language network (**B**, pink) are shown in purple, the tumor is marked in red. The additional anatomical seed region for AF (**C**), IFOF (**E**), ILF (**G**) and UF (**I**) is additionally highlighted by arrows. Reconstructions of AF (**D**, turquoise), IFOF (**F**, orange), ILF (**H**, purple) and UF (**J**, green) in reference to the complete language network are shown in the preliminary form before artifacts were removed manually. Of note, the ILF does not stand out from the whole language network within the present view (**H**). The final tractography for this patient case, after artifacts were removed, can be seen in Fig. 4 (SIA_T)

All DTI data was eddy current and distortion corrected with a system-integrated algorithm on the basis of elastic deformation of the distorted B0 images in reference to the anatomic T1-weighted image sequence (Hiepe 2017; Gerhardt et al. 2019; Coenen et al. 2021). This approach relies on an automatic image segmentation on the basis of a synthetic tissue model (patent WO 2014063840 A1) and subsequent semi-elastic image fusion and co-registration of subdivided three-dimensional image volumes (Hiepe 2017; Gerhardt et al. 2019). The system-integrated fiber assignment continuous tracking and tensor deflection algorithms (Weinstein et al. 1999; Mori and van Zijl 2002; Mori et al. 1999) were used to create the final tractographies of the complete language network as well as specific language tracts. Based on previous studies which systematically assessed the optimal parameters for nTMS-based tractographies of crucial language tracts, all nTMS-positive sites with rims of 5 mm were taken as seed regions to derive the complete functionally relevant left-hemispheric language production network; the maximal angulation was set to 20^°^, the minimum fiber length to 100 mm, the minimum FA to 0.1 (Sollmann et al. 2020b; Negwer et al. 2017a). Subsequently, on the basis of the complete left-hemispheric functional language network, different individual language pathways were delineated. This approach was described in previous publications to allow the visualization and reconstruction of specific, functional language relevant tracts (Ille et al. 2018; Sollmann et al. 2020a; Negwer et al. 2017a). Moreover, this approach showed to be better suited inter alia for the visualization of AF, ILF, UF and IFOF compared to an anatomic cubic region of interest protocol (Negwer et al. 2017b). By introducing a single additional anatomical region of interest, the reconstruction of the complete functional language network could be broken down into the following separate functional-relevant tracts systematically and reproducibly across patients: AF, IFOF, ILF, and UF. The anatomical areas for the former two were based on Ille et al. (2018) and placed in the external capsule (IFOF) and near the posterior horn of the lateral ventricle (AF) within fibers oriented dorso-rostrally, respectively. For ILF, a region within the white matter between the lateral and circular sulcus of the insula, and UF, an area between the circular sulcus, anterior putamen, and head of nucleus caudates, were marked as additional anatomical seeds based on a protocol proposed by Fekonja et al. (2019). This process is illustrated in Fig. 1 which shows the nTMS positive points as well as the additional anatomical ROI used to reconstruct and identify the complete functional language network as well as isolated language tracts. Thereafter, artifacts, such as single anatomical implausible fiberpath courses or fibers not belonging to the respective fiberpath reconstructed, were removed manually. Figure 1 demonstrates the reconstructions of these language tracts before artifacts were removed, the final tractography results without artifacts—which were used for all subsequent analyses for the same patient case—are depicted in Fig. 4. Based on current literature of anterior, posterior and long AF components, anatomically correct cortical terminations within the inferior frontal or ventral precentral gyrus; superior, posterior middle and inferior temporal gyrus as well as the angular and supramarginal gyrus were accepted for the AF (Forkel et al. 2020; Catani and Mesulam 2008; Ivanova et al. 2021; Martino et al. 2013). Since anatomic dissection and DTI studies showed frontal, temporal and parietal endpoints for superficial as well as deep IFOF subcomponents (Martino et al. 2010; Sarubbo et al. 2013; Vassal et al. 2018), cortical terminations within inferior and middle frontal gyrus, the dorso-lateral prefrontal and orbito-frontal cortex as well as frontal pole; and occipital extra-striate cortex as well as superior parietal lobule were accepted for the IFOF. For ILF, cortical terminations within occipital extra-striate cortex and anterior temporal areas were considered as anatomically correct (Latini et al. 2017; Catani et al. 2003). Cortical terminations within anterior temporal lobe as well as orbitofrontal cortical sites were accepted as anatomically correct cortical terminations of UF (Catani et al. 2013a).

Subsequently, the following qualitative and quantitative tract properties were extracted: First, whether a functionally relevant language tract was visualizable pre- and post-craniotomy was assessed. Second, the mean tract-specific FA and tract volume relative to the individual volume of the functional language network was analyzed again for the pre-and postoperative status. For this, automatically calculated average FA values of the reconstructed tracts were extracted within Brainlab Elements (Brainlab AG, Germany). Additionally, each of the reconstructed tracts was defined as an object within the Brainlab Elements Fibertracking module (Brainlab AG, Germany) which allowed to derive the volume of each tract and the complete reconstruction of the left-hemispheric language network in cm^3^. Third, we normalized the volume of each tract by the total volume of the dissected functional left-hemispheric language network by calculating the relative volume of a particular tract in relation to the individual whole language network volume to account for inter-individual differences between patients. Moreover, the percentage of volume loss at the postoperative time point compared to the preoperative state was analyzed descriptively. Fourth, the lesion-to-distance in mm (LTD) was defined in the preoperative contrast-enhanced T1-weighted gradient echo sequence by measuring linearly the minimal distance between tumor mass and closest fibers of a corresponding language tract (Sollmann et al. 2020a). Finally, a recently published approach by Tuncer et al. (2021) was followed to evaluate surgery-related damages of these language-relevant tracts. To this end, the preoperative tractography was fused with the postoperative structural MR images, and distortion correction was applied to minimize potential misalignments caused by brain shift. If a preoperative tract and the resection cavity overlapped, the resection cavity and tract intersection overlap volume in cm^3^ was calculated (RTIOV).

### Classification of surgery-induced language deficit

The type and severity of aphasia were rated with a previously published grading system adapted from the Aachener Aphasia Test (Ille et al. 2016a; Picht et al. 2013; Huber et al. 1983). Based on this, non-fluent and fluent aphasia were differentiated. The patient’s language abilities were assessed during the clinical routine before surgery, on the fifth day postoperatively and three months postoperatively.

Subsequently, patients were assigned to four different groups:

1. Patients without pre- and post-operative aphasia (no aphasia group, NoA)
2. Patients who present with a pre-existing persisting aphasia either caused by the tumor or a previous resection before this surgery, which does neither worsen nor improve after the present surgery (TIA_P)
3. Patients with transient surgery-induced worsening of language abilities, i.e., worsened language skills directly post-surgery, resolved at the three-month follow-up examination (SIA_T)
4. Patients with permanent surgery-induced worsening of language abilities still present at the three-month follow-up examination (SIA_P)

### Statistical analysis

R version 3.6.3 (R Core Team 2022) was used for statistical analyses. A p-value < 0.05 was considered statistically significant. Chi-square or Fisher’s exact tests were computed to compare the frequency of visualizable tracts per group. For all other quantitative variables, a one-way between-subjects ANOVA or, if its assumptions were not met, a Kruskal–Wallis test was used to compare the four groups. All ANOVAs indicating a significant group difference were followed with a Tukey’s HSD Test for multiple comparisons. If a Kruskal–Wallis test showed a significant result, a post-hoc Dunns-Bonferroni test was used to determine which groups differed significantly.

## Results

### Patient and tumor characteristics

This study included the nTMS language mapping results of 74 patients with a mean age of 53.3 ± 15.9 (range 20–81) years, of whom 41.9% were female and 58.1% male. The most significant proportion of these patients was right-handed (88.1%), 7.5% were left-handed, and 4.5% were ambidextrous according to the Edinburgh handedness inventory (Oldfield 1971); seven patients reported no handedness. Most of the patients presented with a WHO CNS grade 3 (20.3%) or grade 4 glioma (66.2%), for only 4.1% a WHO CNS grade 1 and 9.5% a WHO CNS grade 2 glioma was confirmed by histopathology. Tumor locations comprised perisylvian left-hemispheric areas considered language-eloquent according to a classification by Ille et al. (2021a). With a median language eloquence of 6, the present sample showed a high language eloquence (high: 56.8%, moderate: 39.2%, low: 4.1%). While for all patients, the functional status at all time points and the preoperative tractography results could be investigated, for 32 patients, no postoperative diffusion sequences were available. Thus, this study evaluated whether tracts were visualizable postoperatively, the postoperative mean tract-specific FA, and relative tract volume for 42 patients.

### Language status

In 23 cases, neither preoperative nor postoperative aphasia manifested, whereas 51 patients showed aphasic symptoms. Of these, 88.2% were classified as non-fluent aphasia, while only 11.8% were diagnosed as fluent. Due to these limited numbers of patients with fluent aphasia, no comparisons between the two aphasia types were feasible within the present cohort. Next to the 23 NoA cases, 25 patients presented with TIA_P, 22 patients with a transient worsening resolved at the three-month follow-up (SIA_T), and four patients with permanent aphasia after surgery (SIA_P). The patient characteristics per group are summarized in Table 1. The groups did not differ significantly in age [F (3, 70) = 0.12, p = 0.95]. Moreover, Fisher exact tests did not reveal a significant association of aphasia type with sex (p = 0.30), WHO CNS grade (p = 0.10), or fluency (p = 0.64).

**Table 1:** Overview of patient and tumor characteristics per aphasia group

	Aphasia type			
	NoA	TIA_P	SIA_T	SIA_P
Sex (in %, female/male)	43.5/ 56.5	28.0/ 72.0	54.5/ 45.5	50.0/ 50.0
Age (in years, mean ± SD [range])	52.8 ± 18.7 [20–79]	52.6 ± 14.4 [25–77]	55.2 ± 16.1 [23–81]	54.3 ± 9.8 [42–64]
WHO CNS Grade (in %)				
1	4.3	0.0	9.1	0.0
2	4.3	4.0	22.7	0.0
3	26.1	24.0	4.5	50.0
4	65.2	72.0	63.6	50.0
Fluency of aphasia^a^ (in %)				
f	0.0	8.0	18.2	0.0
n-f	0.0	92.0	81.8	100.0

^a^Fluent (f) and non-fluent (n-f) aphasia

### Results of function-specific tractography

#### Feasibility of reconstructing language tracts

**Fig. 2:**
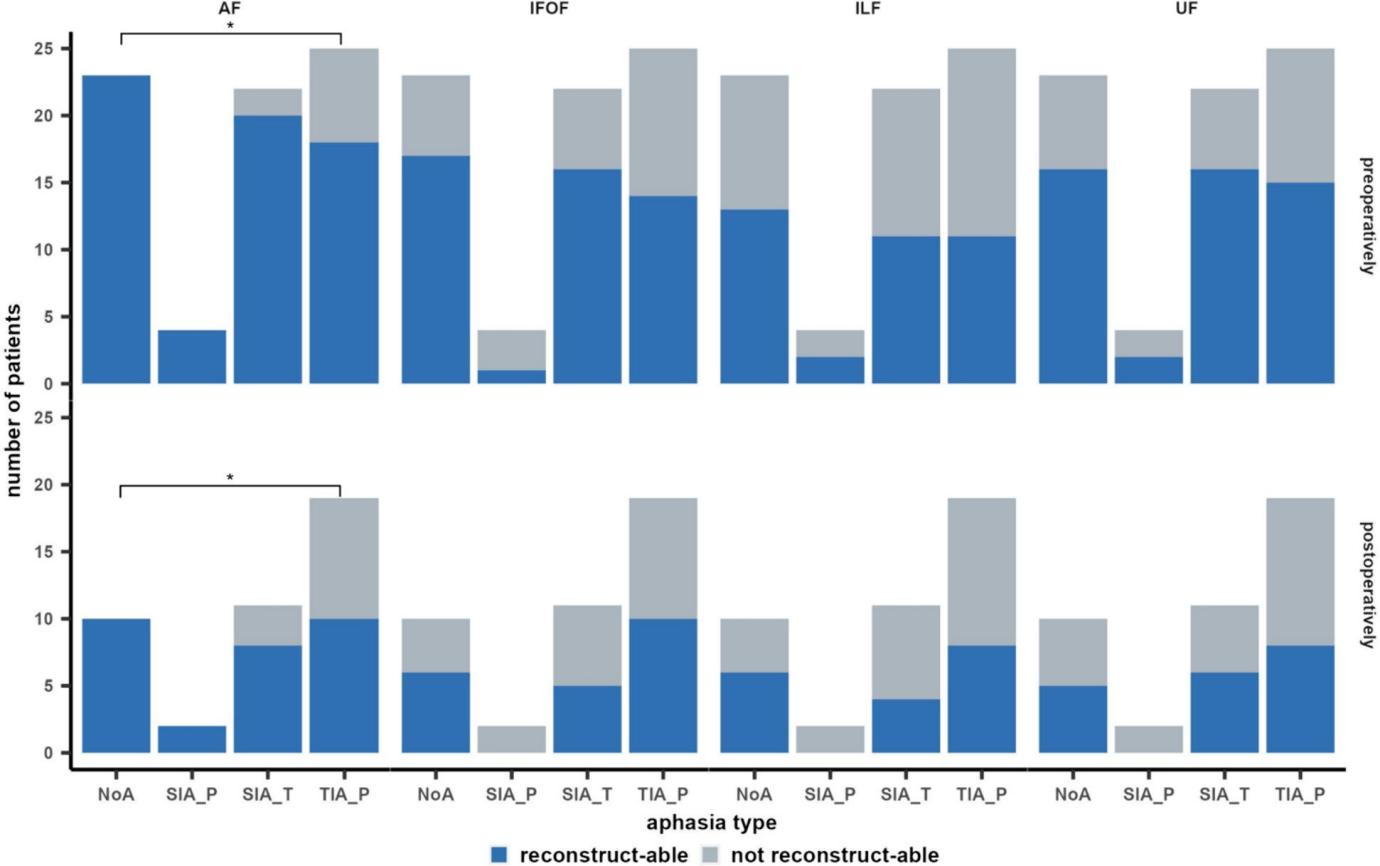
Overview of the number of patients for whom AF, IFOF, ILF, and UF were visualizable (blue) compared to those for whom these tracts could not be visualized (grey) pre-and postoperatively. *p < 0.05, ** p < 0.01, *** p < 0.001

Functionally relevant AF and IFOF could be visualized in many patients pre- and postoperatively (Table 2). ILF and UF could be reconstructed in comparatively fewer patients based on the nTMS results and the tractography parameters. Statistical analyses revealed no significant differences in the reconstructed tract frequency at each time point between SIA_T nor SIA_P with any other aphasia type. Moreover, statistical analyses did not show significant group differences in the frequency of patients for whom IFOF, ILF, or UF could be reconstructed. Of note, primarily anterior components of the UF were visualizable; in most cases, the complete UF could not be reconstructed (Fig. 4). The results of Fisher’s exact test indicated that AF was significantly more frequently visualizable in patients without any aphasia compared to patients with TIA_P pre- and postoperatively (both p = 0.01). Figure 2 illustrates the frequency of patients for whom each of the four language tracts could be reconstructed pre-and postoperatively.

**Table 2:** Overview of qualitative and quantitative tract properties of functional AF, IFOF, ILF, and UF for each aphasia type

	Aphasia type			
	NoA	TIA_P	SIA_T	SIA_P
n patients (pre-/postoperatively)	23/10	25/19	22/11	4/2
Visualizable tract (in %, pre-/postoperatively)				
AF	100.0/100.0	72.0/52.6	90.9/72.7	100.0/100.0
IFOF	73.9/60.0	56.0/52.6	72.7/45.5	25.0/0.0
ILF	56.5/60.0	44.0/ 42.1	50.0/36.4	50.0/0.0
UF	69.6/50.0	60.0/ 42.1	72.7/54.5	50.0/0.0
Relative tract volume (mean ± SD; pre-/postoperatively)				
AF	0.33 ± 0.21/0.29 ± 0.18	0.41 ± 0.25/0.42 ± 0.33	0.26 ± 0.14/0.33 ± 0.20	0.30 ± 0.18/0.26 ± 0.17
IFOF	0.07 ± 0.04/0.12 ± 0.07	0.11 ± 0.10/0.09 ± 0.07	0.09 ± 0.07/0.07 ± 0.06	0.07 ± NA/NA
ILF	0.07 ± 0.06/0.06 ± 0.06	0.08 ± 0.07/0.04 ± 0.02	0.04 ± 0.04/0.03 ± 0.02	0.04 ± 0.02/NA
UF	0.06 ± 0.04/0.10 ± 0.07	0.05 ± 0.04/0.11 ± 0.08	0.06 ± 0.04/0.07 ±0.05	0.01 ± 0.00/NA
RTIOV (in cm^3^, mean ± SD)				
AF	0.1 ± 0.1	0.4 ± 0.8	0.4 ± 0.6	0.6 ± 0.7
IFOF	0.0 ± 0.1	0.2 ± 0.3	0.1 ± 0.3	0.0 ± NA
ILF	0.0 ± 0.0	0.0 ± 0.0	0.1 ± 0.2	0.2 ± 0.2
UF	0.0 ± 0.0	0.0 ± 0.0	0.0 ± 0.0	0.0 ± 0.0
LTD (in mm, mean ± SD				
AF	10.6 ± 11.1	7.6 ± 11.6	6.5 ± 6.4	5.1 ± 6.7
IFOF	9.4 ± 10.0	10.3 ± 10.6	14.4 ± 15.7	23.0 ± NA
ILF	19.2 ± 16.5	14.8 ± 14.5	21.4 ± 13.0	10.5 ± 14.8
UF	24.2 ± 22.5	29.6 ± 17.3	28.8 ± 15.7	18.5 ± 26.2
FA (mean ± SD; pre-/postoperatively)				
AF	0.39 ± 0.05/0.36 ± 0.04	0.35 ± 0.08/0.36 ± 0.07	0.38 ± 0.07/0.39 ± 0.07	0.36 ± 0.05/0.41 ± 0.03
IFOF	0.36 ± 0.05/0.32 ± 0.04	0.30 ± 0.06/0.31 ± 0.05	0.38 ± 0.05/0.33 ± 0.06	0.40 ± NA/NA
ILF	0.38 ± 0.04/0.35 ± 0.04	0.30 ± 0.06/0.31 ± 0.05	0.32 ± 0.04 /0.32 ± 0.04	0.32 ± 0.03 /NA
UF	0.36 ± 0.07/0.31 ± 0.03	0.31 ± 0.09/0.30 ± 0.08	0.37 ± 0.07/0.32 ± 0.07	0.31 ± 0.09/NA

#### Tract-specific volume

The descriptive statistics of the relative tract-specific volumes for each of the four groups are summarized in Table 2. The relative volume of AF, IFOF, ILF, and UF did not differ significantly between the four aphasia types (all p ≥ 0.29).

The largest loss in AF volume was observed for the SIA_T group (−61.3%). Still, the NoA (−57.7%) and TIA_P (−53.0%) showed a comparable loss in AF volume. At the same time, the SIA_P group had the lowest AF volume loss (-45.6%). Still, 100.0% of IFOF, ILF, and UF volume were lost postoperatively, as these could not be reconstructed for any SIA_P cases (see Fig. 2). The NoA cases showed comparable UF (− 59.9%) and AF volume loss, as well as a loss of − 44.2% in IFOF and − 7.0% in ILF volume. The transient aphasia group showed a loss of −54.5% in UF, −40.4% in ILF, and −18.5% in IFOF volume. Although the TIA_P group showed an ILF volume loss of −43.6%, the postoperative volume of IFOF exceeded the preoperative by 104.6% and the one of UF by 26.5%.

#### Overlap volume of resection cavity and language tract intersection

No significant differences in RTIOV for IFOF (p = 0.78), ILF (p = 0.15), and AF (p = 0.09) between groups were observed. While the UF had a distance of 0 mm to the tumor in isolated cases, no proportion of this tract overlapped directly with the resection cavity across groups. Consequently, within the present cohort, no fraction of the UF was resected during craniotomy. Moreover, the preoperative ILF overlapped for none of the NoA cases, and the preoperative IFOF for none of the SIA_P cases with the respective resection cavity.

#### Lesion-to-tract distance

The four aphasia groups did not differ significantly in the distance between the lesion and any functional language tracts (all p ≥ 0.52). The mean and standard deviation for each tract per aphasia type pre- and postoperatively are summarized in Table 2.

#### Tract-specific fractional anisotropy

**Fig. 3:**
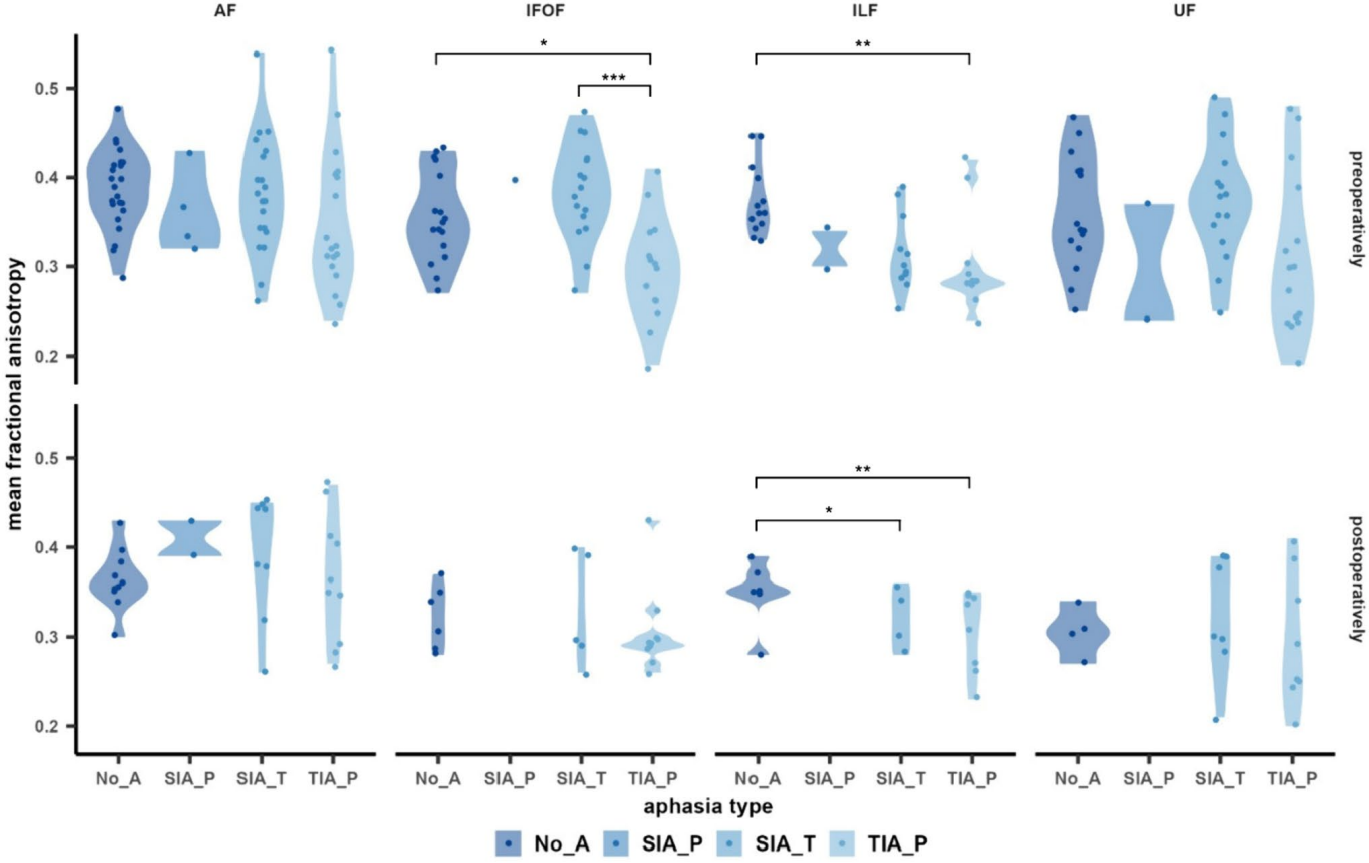
Violin plots illustrating the mean fractional anisotropy of functional AF, IFOF, ILF, and UF for each of the four groups pre-and postoperatively. *p < 0.05, ** p < 0.01, *** p < 0.001

The mean FA for each functionally relevant language tract was compared across groups. A descriptive overview is provided in Table 2. A one-way ANOVA revealed a significant difference between groups for the FA of IFOF preoperatively (F (3, 44) = 6.6, p < 0.001). Tukey’s HSD test for multiple comparisons showed that the mean IFOF fractional anisotropy was significantly lower in the TIA_P compared to the NoA group (p = 0.03) and compared to the SIA_T group (p < 0.001) preoperatively (Fig. 3). Moreover, the preoperative FA of the ILF was associated with the type of aphasia (χ^2^ = 13.3, p < 0.01). Post hoc Dunn-Bonferroni tests showed that the NoA group had significantly higher mean FA than the TIA_P group (p < 0.01) (Fig. 3). The FA of AF and UF did not differ between groups preoperatively. Moreover, the only language tract for which statistical analyses revealed significant group differences in the FA postoperatively was the ILF (F (3, 33) = 5.78, p < 0.01). Post-hoc Tukey’s HSD verified a significantly higher FA for the NoA compared to the SIA_T (p = 0.02) and TIA_P group (p < 0.01), respectively.

#### Descriptive analysis of surgery-induced permanent aphasia

Since only four patients presented with SIA_P, it was expected that no statistically significant group-wise differences could be found within the present cohort. Still, identifying patients at risk of permanent postoperative deterioration of language functionality is a crucial objective during resections of language-eloquent brain tumors. Consequently, this group’s qualitative and quantitative tractography results were compared descriptively to the other three aphasia types. The exact numbers are summarized in Table 2. These results indicated that SIA_P cases had the highest mean RTIOV of AF, the shortest mean AF to lesion distance, the lowest mean relative AF volume post-surgery, and the highest mean AF fractional anisotropy postoperatively. Moreover, only a single SIA_P case had a visualizable IFOF before surgery. As for this patient, no postoperative DTI was available, the IFOF status could not be evaluated post-surgery.

## Discussion

The present study compared qualitative and quantitative pre- and postoperative nTMS-based tractography properties in glioma patients with and without aphasia to identify suitable pathophysiological indicators of permanent or transient language deficits. Function-based pathophysiological risk stratification may be derived by evaluating and combining the reconstruction ability, tract-specific FA, LTD, volume (loss), and RTIOV.

### Quantifying surgery-induced structural tract impairments

DTI tractography is one of the most frequent and well-integrated imaging methods to study white matter connections in research and in the preoperative neurosurgical setting (Tae et al. 2018; Caverzasi et al. 2016). For instance, Reisch et al. (2022) proposed to use anatomical-guided DTI tractography of the AF to guide the selection of stimulation targets during nTMS-based language mappings. While this may support a more accurate identification of cortical endpoints of a specific anatomical language tract, other cortical, language relevant sites may be missed as the language network spreads over a multitude of cortical sites and subcortical streamlines. An alternative approach, which was also implemented within the present study, is to use a standard protocol based on anatomical cortical landmarks for nTMS stimulation target identification within frontal, parietal and temporal areas, and subsequently use the language-relevant cortical sites as seeds for DTI tractography. By performing nTMS-guided DTI language tractography, the functional language network and specific functional language tracts can be reconstructed (Raffa et al. 2016; Negwer et al. 2017a). Negwer et al. (2018) demonstrated that the reconstruction ability of AF post-surgery can indicate postoperative deficits. While non-visualizing a tract with DTI tractography may reflect a structural impairment caused by surgery or the resection of a tract, it may also be confounded by edema, spurious fibers, or lowered FA caused by the tumor itself or fiber distortions (Jung et al. 2014; Negwer et al. 2018). Although the causes for non-visible AF are heterogeneous, the present study showed that the AF cannot be reconstructed in more patients with TIA_P compared to NoA patients, indicating a link between structural tract impairment and aphasia. Moreover, across SIA_T cases, the AF reconstruction ability was slightly reduced before surgery and could only be reconstructed in 73% postoperatively. Hence, this factor may be suitable for stratification of the risk before surgery and informing rehabilitation approaches directly post-surgery. Another study by Ille et al. (2018) quantified the loss by comparing the change in the number of fibers of a functional language tract over time. Similar to this approach, the present study compared the loss in tract volume pre-and postoperatively. Current NoA cases showed a volume loss across all four language tracts, while for TIA_P cases, a gain in UF and IFOF volume was observed directly postoperatively. The latter may be explained by a postoperative reduction in edema and, consequently, a decrease in DTI artifacts, leading to a higher volume of fibers to be reconstructed. The present SIA_P cases showed a complete volume loss of all ventral language tracts directly post-surgery. Previous research related a loss directly postoperatively and a re-gain of fibers several months post-surgery with SIA_T (Ille et al. 2018). Thus, postoperatively comparing these tract properties across multiple time points may provide even more accurate neural correlates and diagnostic indicators of the patient’s functional status. Another direct way to quantify the injury of specific language pathways is the calculation of RTIOV (Tuncer et al. 2021). Since SIA_P cases had the highest AF RTIOV (Table 2), it may be a suitable factor to quantify the risk of permanent postoperative aphasia.

### Quantifying the preoperative aphasia risk

Another quantitative factor frequently evaluated in the context of aphasia risk stratification is LTD. Previous studies linked short proximity between AF and the tumor to more severe or permanent language deficits and a higher mortality (Meyer et al. 2017; Li et al. 2020; Sollmann et al. 2020a). Although all these studies suggested specific cut-off AF LTD criteria to identify patients at risk of postoperative aphasia, the proposed value ranged from 3.2 to 15.4 mm. Unlike these studies, the LTD did not significantly differ between the groups for any of the four assessed language tracts within the present cohort. Still, descriptive results linked the SIA_P group to the shortest AF LTD, in line with previous findings. The small sample size of the SIA_P group may explain the non-significant group differences. Within the present sample, SIA_P cases had, on average, only an AF LTD of 5.1 mm. Across all groups, the average AF LTD was lower than 15.4 mm. Hence, the current results suggest that the cut-off criteria proposed by previous studies could not be applied to this sample. Extensive multi-centric studies are warranted to define a generalizable cut-off value for AF LTD. DTI, moreover, allows the analysis of microstructural tissue properties. Alterations in axonal integrity, as reflected by FA, have been associated with ample neurological and neurodegenerative disorders (Tae et al. 2018). A recent study linked reduced preoperative IFOF, UF, and ILF integrity to permanent aphasia (Prasse et al. 2023). In line with these results, the current study demonstrated significantly reduced preoperative IFOF integrity in patients with TIA_P compared to NoA and SIA_T. The non-significant differences postoperatively may be explained by the limited number of patients for whom IFOF could still be reconstructed postoperatively and may have been driven by a single TIA_P case who showed a postoperative IFOF FA value exceeding the ones of the NoA and SIA_T groups (Fig. 3). Additionally, ILF integrity was reduced in TIA_P cases compared to patients without aphasia pre- and postoperatively and in SIA_T compared to NoA cases postoperatively. IFOF and ILF have been associated with aphasia in tumor patients (Southwell et al. 2017; Ius et al. 2011; Sarubbo et al. 2020; Tuncer et al. 2021). The present results show that microstructural tissue properties such as the FA can be used to quantify reduced axonal integrity in these ventral language streams. However, these results must be interpreted cautiously as perilesional edema can decrease FA, confounding the evaluation of a tract’s integrity (Prasse et al. 2023).

### Significance of the present study

The present study linked predominantly qualitative and quantitative functional AF and IFOF properties derived from nTMS-based tractographies to aphasia in brain tumor patients. That these two tracts play pivotal roles in language functionality and respective injuries for the development of language impairments in brain tumor patients is well established (Tuncer et al. 2021; Ius et al. 2011; Sarubbo et al. 2020; Duffau 2015; Caverzasi et al. 2016). At the same time, damage to the indirect ventral pathway components, i.e., UF and ILF, may be compensable (Duffau et al. 2009; Ius et al. 2011). This is supported by the present findings, which showed a predominant association of qualitative and quantitative dorsal and direct but not indirect ventral stream components with the expression of aphasia in glioma patients. Since it is assumed that the ventral and dorsal stream subserve distinct language processing steps (Hickok and Poeppel 2004; Duffau et al. 2014), subsequent studies may evaluate whether these function-guided quantitative and qualitative tract properties are even predictive of the type of linguistic impairment. Previous studies, for instance, linked the (inferior ventral) AF and (middle and posterior) IFOF to language comprehension (Ivanova et al. 2016; Shams et al. 2023). At the same time, the ILF or anterior sub-components were associated with language production (Ivanova et al. 2016; Shams et al. 2023). Still, results across studies differ and language production and comprehension were not always associated with the same or any specific language tract damages (Billot et al. 2022). Since most of the patients within the present cohort showed non-fluent deficits, no group-wise comparisons between aphasia subtypes were feasible within the present study. Thus, further studies are warranted to evaluate whether specific language network subcomponents contribute to a specific language function and can provide preoperative indicators of the type of impairment to expect postoperatively.

Overall, the present results suggest that different qualitative and quantitative functional tract properties such as reconstruction ability, relative tract-specific volume, the RTIOV, its LTD, and mean FA may support the differentiation of patients without aphasia, with persisting aphasia caused by a previous resection or the tumor itself, and new postoperative transient or permanent language deterioration. Next to stratifying the risk of aphasia preoperatively, these pathophysiological properties may guide preoperative prehabilitation and postoperative language rehabilitative therapeutic approaches. While studies assessing the suitability of such stimulation-based interventions to support the preservation or recovery of language functionality by actively promoting functional reorganization processes in heterogeneous lesion populations are accumulating, identification of optimal stimulation targets remains challenging (Einstein et al. 2022; Shah et al. 2013; Torres et al. 2013). Hence, these qualitative and quantitative functional tract properties may aid patient-specific target definition for stimulation-based interventional approaches tailored to the individual pathophysiological profile.

### Limitations and perspectives

While the number of patients included in this exploratory study (n = 74) exceeded many studies on risk stratification in brain tumor patients (Tuncer et al. 2021; Ille et al. 2018; Prasse et al. 2023; Li et al. 2020; Meyer et al. 2017), the limited sample size of patients with SIA_P (n = 4) impacted the statistical results and confounded generalizability. Although descriptive results indicated that the examined qualitative and quantitative functional tract properties, particularly of left-hemispheric AF, may be suitable for assessing the risk of permanent language deterioration post-surgery, subsequent studies with a larger sample size must confirm this first indication.

Moreover, some limitations must be considered when interpreting the results of DTI tractography. A deterministic tractography algorithm was employed following previous protocols for language tractography based on nTMS language mapping (Sollmann et al. 2020b; Negwer et al. 2017a). Imaging and tracking parameters and algorithms directly impact the tractography results (Caan 2016; Le Bihan et al. 2006). Moreover, DTI tractography is prone to potential false positive and false negative representations of fiberpaths (Maier-Hein et al. 2017; Aydogan et al. 2018; Grisot et al. 2021; Campbell and Pike 2014; Catani et al. 2013b). Thus, the tensor-based tractography approaches may inaccurately represent the language tracts. A decade ago, it was frequently argued that DTI-based results are not reliable enough for the neurosurgical setting (Farquharson et al. 2013; Duffau 2014). Therefore, the pre- and intraoperative use of DTI-based reconstructions of networks remains controversial across centers. Still, more and more advanced pre-processing, tractography and alternative approaches, such as high angular resolution diffusion or diffusion spectrum imaging, or constrained spherical deconvolution became available to account for the limitations of DTI (Henderson et al. 2020). Yet, DTI remains the method of choice for preoperative pathway visualization and surgical planning in the neurosurgical treatment of brain tumors (Henderson et al. 2020). A recent meta-analysis indicated an even higher rate of gross total resection for DTI-based neurosurgeries than the gold standard, intraoperative subcortical monitoring, and a comparable postoperative functional outcome (Li et al. 2023). Additionally, previous work demonstrated that nTMS-based tractography approaches such as the one used in this study can guide neurosurgical resections of brain tumors with comparable functional outcomes as cortical and subcortical direct electrical stimulation (Ille et al. 2021a). This supports the utility and validity of these functional network reconstructions.

Thus, it is crucial to identify clinically applicable and easily integrable factors for risk stratification based on DTI tractographies. At the same time, heterogeneous hardware, imaging, and tractography parameters are employed across different studies, complicating the direct comparisons of results across centers and the definition of a generalizable cut-off criterion. All analyses and results presented within the present study were derived with Brainlab Elements (Brainlab AG, Germany). Thus, it remains unclear whether the present results are generalizable if a different software and different DTI sequences are used. Still, Brainlab Elements is a software which is frequently used within the clinical preoperative neurosurgical settings across multiple centers and offers a workflow which is easily integrable into the preoperative planning as well as the intraoperative neuronavigational process. Since the primary aim of the present study was to identify diagnostic indicators for preoperative risk stratification which may be integrated into the preoperative workflow to aid patient consultation and subsequent interventional decisions, this closed system was chosen as opposed to alternative, freely available DTI analysis toolboxes primarily used for research.

**Fig. 4:**
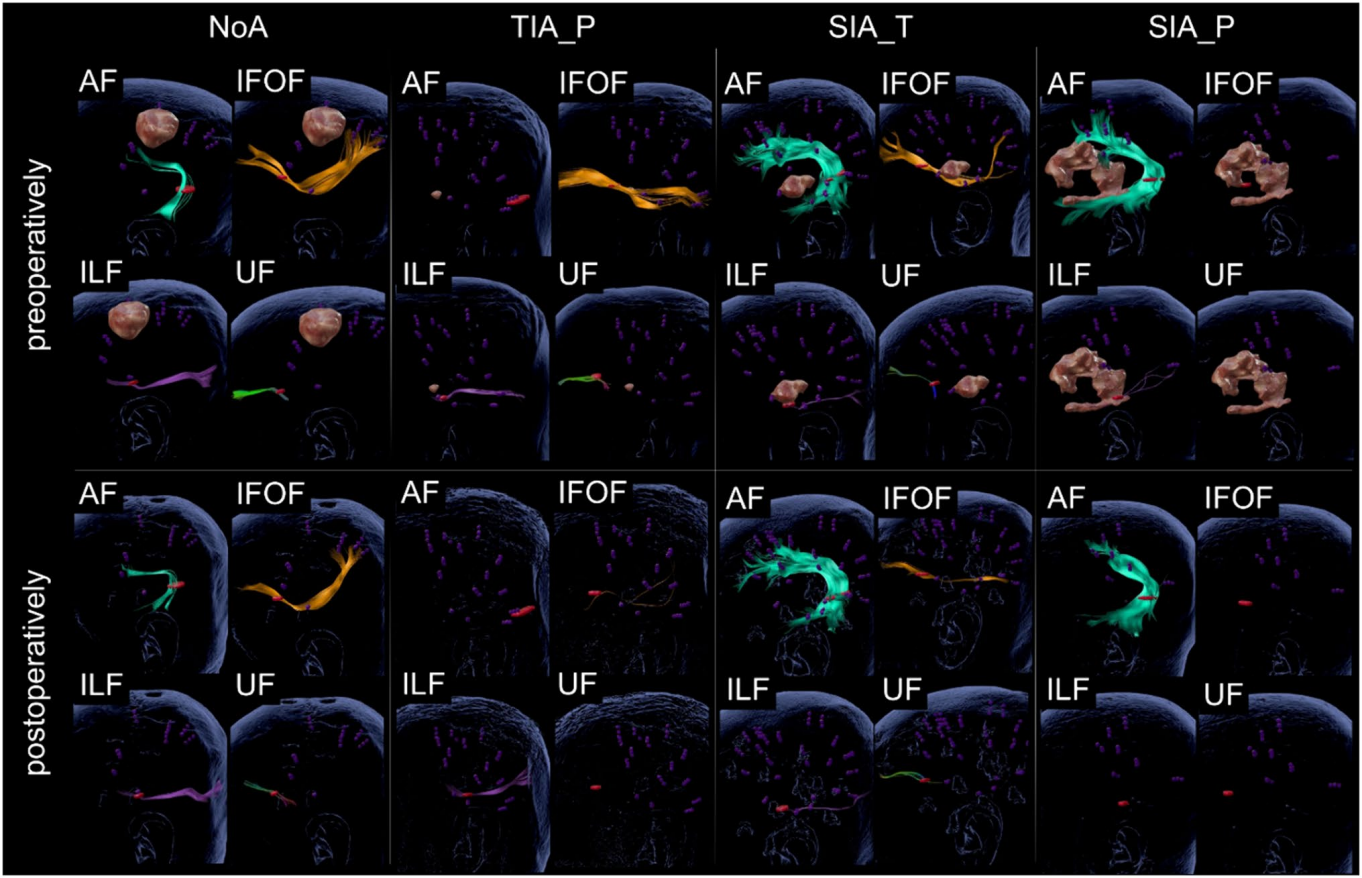
Illustrative tractographies of functional language tracts (AF in turquoise, IFOF in orange, ILF in purple, UF in green) for each aphasia type pre-and postoperatively. The respective tumor is highlighted in brown within the preoperative tractographies

Additionally, since the individual nTMS mapping results were used as seed regions for the reconstruction of functional language tracts, the seed regions across patients varied which may impact inter-patient comparability. Still, the primary aim of the present study was to reconstruct and compare parameters of the functional language network for each individual patient. Since inter-individual variability is expected at single case level, more individualized approaches such as the one followed in the present study, may be necessary to evaluate the functional language network as opposed to the structural one. Nevertheless, to allow for inter-patient comparisons, the same tractography parameters were applied across patients and the same additional anatomical region of interest used. To additionally account for inter-individual variability, the relative tract volumes were calculated and compared between groups. Furthermore, nTMS-based language mappings are prone to false positive language mapping results compared to the gold standard, direct electrical stimulation during awake surgery (Bährend et al. 2020; Picht et al. 2013; Tarapore et al. 2013; Ille et al. 2015). However, studies indicated nTMS-based language mapping and subsequent nTMS-based DTI tractography results allow for reliable reconstructions of the functional language network, supporting the preservation of functionality (Raffa et al. 2022; Ille et al. 2016b). Moreover, the present study ascertained only a single microstructural DTI metric. While FA constitutes the most widely applied DTI metric, evaluation of the mean, longitudinal, or perpendicular water molecule displacement (Curran et al. 2016) may support the microstructural identification of patients at risk of aphasia. In addition, no volume threshold cut-off value was used within the present study. Systematically defining a minimum volume as a cut-off value for identifying non-visualizable tracts may allow for an even clearer classification of visualizable and non-visualizable pathways. Since, however, no cut-off value was defined within previous studies for this nTMS-based tractography approach, tracts were only classified as non-visualizable if no fibers for a respective language tract could be identified. Simultaneously, this resulted in the identification of language tracts which in some instances only comprised one to ten streamlines (see e.g. Fig. 4). Hence, it remains unclear, whether these represent proper language tracts. Subsequent studies are warranted which systematically evaluate and define a volume cut-off value. Moreover, the predefined setting of a minimal streamline length of 100 mm may have impacted the number of non-tractable pathways. Still, the current settings were chosen based on previous systematic studies which indicated the settings applied as ideal for visualizing the different language tracts during nTMS-based language tractography within Brainlab Elements (Brainlab AG, Germany) (Sollmann et al. 2020b; Negwer et al. 2017a). Furthermore, this study focused only on left-hemispheric functional language tract properties since nTMS language mapping data was primarily available for the left hemisphere across these left-hemispheric tumor cases. Still, tumors within the language network can prompt functional reorganization processes, which may recruit the non-dominant right hemisphere (Nieberlein et al. 2023). However, whether this right-hemispheric recruitment for language processes is beneficial or maladaptive and whether stimulation-based language rehabilitation should support or inhibit this reallocation remains controversial (Chrysikou and Hamilton 2011; Shah et al. 2013; Turkeltaub 2015). Thus, subsequent studies could opt for bi-hemispheric language mapping and tractography to advance the understanding of the role of the non-dominant right hemisphere for language in lesioned populations.

## Conclusion

Based on nTMS language mapping and DTI tractography of major left-hemispheric language tracts, qualitative and quantitative functional-relevant tract properties could be derived, which allows for differentiation of patients without any, with tumor or previous resection caused persistent and transient or permanent surgery-induced aphasia. Lesion to tract distance and the preoperative reconstruction ability of AF or FA of IFOF provided indications of the type of aphasia before a resection. At the same time, the volume loss of AF and IFOF and the intersection overlap of preoperative AF with the resection cavity were suitable correlates of surgery-induced transient and permanent aphasia. Hence, by combining pre- and postoperative qualitative and quantitative functionally relevant AF and IFOF properties, a feasible and easily integrable tool for individual pathophysiological risk stratification and identifying individual, effective targets for stimulation-based pre- or rehabilitative approaches may be derived. This may substantially support decisions on the surgical approach and the intervention strategy, as well as aphasia prevention and rehabilitation.

## Data Availability

Due to privacy restrictions of our clinical data, individual MRI, DTI, and video data cannot be made publicly available. All data presented in this study are available upon reasonable request.

## Appendix A

The following Figure shows an exemplary fusion of the pre- (yellow outline) and postoperative (blue outline) T1-weigthed sequence which was performed as an additional qualitative control step to check the localization of the nTMS-positive points within the postoperative structural image sequence and control for any inaccuracy, e.g. caused by brain shift. The anatomical accuracy was assessed within the coronal (A), axial (B) and sagittal view (C). The nTMS-positive points are outlined in purple, additional arrows are provided to highlight the exact location of different nTMS positive sites.
